# Persistent immunity after therapeutic vaccination targeting HER2/neu correlated with post-vaccination magnitude of interferon gamma Elispot responses

**DOI:** 10.1186/2051-1426-2-S3-P53

**Published:** 2014-11-06

**Authors:** John B Liao, Jessica L Reichow, Doreen M Higgins, Jennifer S Childs, Lupe G Salazar, Mary L Disis

**Affiliations:** 1 University of Washington, Seattle, WA, USA

## Background

As therapeutic vaccination approaches are explored for cancer therapy, there is a need to identify milestones that can predict the successful induction of sustained immunity. We examined whether the magnitude of immune responses seen prior to vaccination or at the completion of a vaccine series to a tumor-associated self antigen can predict long-term immunity.

## Methods

Interferon gamma Elispot data was abstracted from patients enrolled in 3 clinical trials (n = 86) utilizing therapeutic vaccination targeting HER2/neu with long-term follow-up. A positive response/immunity was defined as a precursor frequency of greater than 1:20,000 or 50 interferon gamma corrected spots per well/10^6 ^peripheral blood mononuclear cells. Responses were evaluated at baseline study entry, at one month after completion of vaccinations and at long-term follow-up, the last time point available at week 24-36. Correlative statistical analysis was performed using Pearson product-moment correlation coefficients of least-squares regression.

## Results

Although the magnitude of immunity at one month post vaccination and at baseline both correlated with immunity at long-term follow-up, immunity at one month post-vaccination has the stronger correlation: (p < 0.0001, r = 0.651) versus (p < 0.0001, r = 0.571). Immunity at one month post-vaccination predicts positivity at long-term follow-up (AUC = 0.7872). Defining a threshold of greater than 400 corrected spots per well at one month post-vaccination yields a 97% certainty that long-term follow-up responses will be positive. The increases from baseline interferon gamma Elispot responses against HER2/neu were also significantly higher in patients that achieved persistent immunity at long-term follow-up time points (p < 0.05).

## Conclusions

The magnitude of interferon gamma Elispot responses from peripheral blood mononuclear cells one month after completion of vaccination correlates best with persistent immunity at long-term follow-up time points. Pre-existing levels of immunity also influence the ability of patients to achieve persistent immunity at long-term follow-up with therapeutic vaccination. This knowledge may be exploited to identify the success of targeted cancer vaccines early and allow the implementation of boosters or the employment of vaccines against alternate targets.

